# Crystal structure of Alzheimer's disease phospholipase D3 provides a molecular basis for understanding its normal and pathological functions

**DOI:** 10.1111/febs.17277

**Published:** 2024-09-26

**Authors:** Kenta Ishii, Stefan J. Hermans, Maria Eleni Georgopoulou, Tracy L. Nero, Nancy C. Hancock, Gabriela A. N. Crespi, Michael A. Gorman, Jonathan H. Gooi, Michael W. Parker

**Affiliations:** ^1^ Structural Biology Laboratory St Vincent's Institute of Medical Research Fitzroy Australia; ^2^ Department of Biochemistry and Pharmacology, Bio21 Molecular Science and Biotechnology Institute University of Melbourne Parkville Australia; ^3^ ARC Centre for Cryo‐electron Microscopy of Membrane Proteins, Bio21 Molecular Science and Biotechnology Institute University of Melbourne Parkville Australia; ^4^ Present address: Monash Institute of Pharmaceutical Sciences Parkville Australia; ^5^ Present address: Research, Innovation and Commercialisation The University of Melbourne Parkville Australia

**Keywords:** Alzheimer's disease, enzyme, nuclease, phospholipase, X‐ray crystallography

## Abstract

Human 5′‐3′ exonuclease PLD3, a member of the phospholipase D family of enzymes, has been validated as a therapeutic target for treating Alzheimer's disease. Here, we have determined the crystal structure of the luminal domain of the enzyme at 2.3 Å resolution, revealing a bilobal structure with a catalytic site located between the lobes. We then compared the structure with published crystal structures of other human PLD family members which revealed that a number of catalytic and lipid recognition residues, previously shown to be key for phospholipase activity, are not conserved or, are absent. This led us to test whether the enzyme is actually a phospholipase. We could not measure any phospholipase activity but the enzyme shows robust nuclease activity. Finally, we have mapped key single nucleotide polymorphisms onto the structure which reveals plausible reasons as to why they have an impact on Alzheimer's disease.

## Graphical abstract

Human 5′–3′ exonuclease PLD3, a member of the phospholipase D family of enzymes, has been validated as a therapeutic target for treating Alzheimer's disease. Here, we have determined the crystal structure of the luminal domain of PLD3 at 2.3 Å resolution. We compare the structure with published structures of other PLD family members and map key single nucleotide polymorphisms onto the structure, which reveal plausible reasons why they impact on Alzheimer's disease.

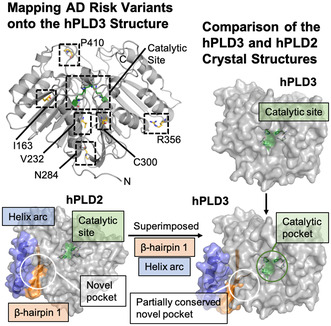

**Abbreviations**
AD: Alzheimer's disease
APP: amyloid precursor protein
ASU: asymmetric unit
CN‐PAGE: clear native page
EFQO: end‐labeled fluorescence‐quenched oligonucleotide
ER: endoplasmic reticulum
PA: phosphatidic acid
PLD: phospholipase D
PLD3: phospholipase D3
PLD1: phospholipase D1
PLD2: phospholipase D2
PLD4: phospholipase D4
SNP: single nucleotide polymorphism

## Introduction

Human phospholipase D3 (hPLD3; EC 3.1.16.1) polymorphisms have been linked to late‐onset Alzheimer's disease (AD). The most clinically represented is the V232 single nucleotide polymorphism (SNP) which is not found in cognitively healthy adults but in AD patients it is associated with accelerated memory decline [1, 2, 3, 4]. AD variants or knockdown of PLD3 are associated with dysregulated amyloid precursor protein (APP) processing and a concomitant increase in extracellular Aβ levels whereas PLD3 overexpression decreases levels of both [1, 5, 6]. APP processing leading to the generation of the neurotoxic Aβ peptide is believed to be a key initiating event of AD. A number of later studies have challenged the veracity of the genetic associations, which may in part be due to technical limitations of studying rare gene coding variants [7, 8, 9, 10, 11]. Landmark papers in the field over the last year provide strong evidence of the involvement of PLD3 in AD. Firstly, amyloid plaque‐associated axonal spheroid enlargement, caused by hPLD3, was shown to cause neural network dysfunction, a feature of AD pathology [12]. Reducing hPLD3 levels reversed axonal conduction abnormalities and restored neuronal network function in an AD mouse model. Secondly, molecular crosstalk between hPLD3, c‐GAS‐STING signaling and APP processing pathways has been revealed [13].

PLD3 is highly abundant in the lysosomes of brain neurons, particularly in the cortex and the hippocampus, both areas that are vulnerable to AD pathology [5, 14, 15]. The physiological function of hPLD3 in neurons and regulation has been controversial [1, 16]. PLD3 is synthesized as a type II membrane protein in the endoplasmic reticulum (ER). It is transported from the ER and Golgi via endosomes to lysosomes, where the cytosolic N‐terminal membrane‐bound domain of full‐length PLD3 is proteolytically cleaved, releasing a 55 kDa soluble luminal PLD3 [14]. PLD3 deficiency affects lysosomal morphology [1, 5, 16].

**Fig. 1:**
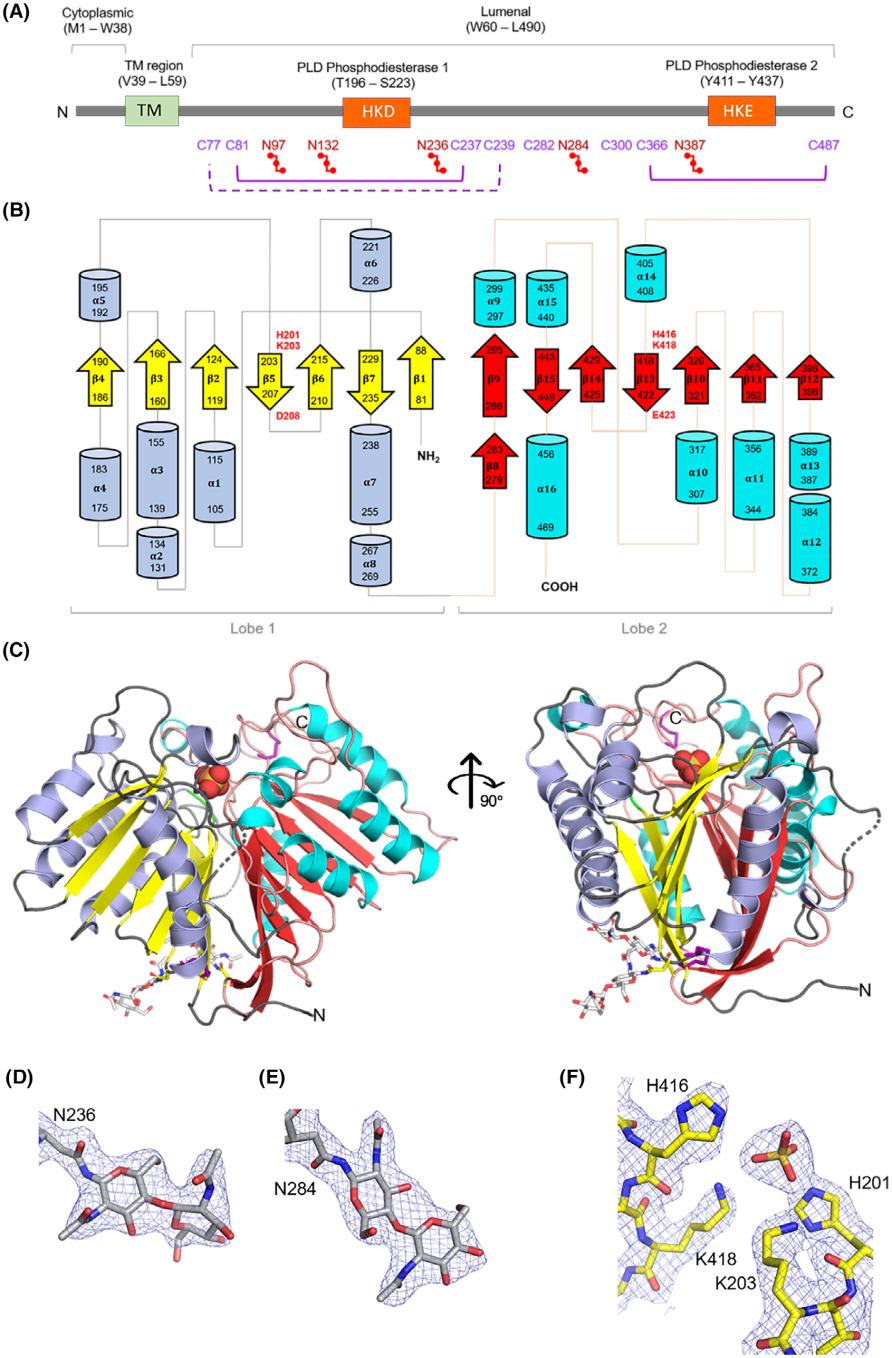
Crystal structure of human PLD3 luminal domain determined at 2.3 Å resolution. (A) Cartoon of domain organization. Full‐length hPLD3 (residues 1–490) consists of a short N‐terminal cytoplasmic domain (M1‐W38), a transmembrane (TM) region (V39‐L59) and a long C‐terminal luminal domain (W60‐L490) containing two PLD phosphodiesterase domains. The HKD and HKE motifs are located in PLD phosphodiesterase domains 1 and 2, respectively. Cysteine residues are color‐coded in purple and the solid lines connecting pairs of cysteine residues indicate a disulfide bond. C77 and C239 do not form a disulfide bond in our structure, as indicated by the dashed purple line; however, this disulfide is observed in the structure reported by Roske *et al*. [29]. Asparagine residues confirmed to be N‐linked glycosylation sites are color‐coded in red with a linker representation [29]. We observed clear density for N‐linked glycosylation (GlcNAc) at N236 and N284. (B) Topological diagram illustrating the secondary structure features in the PLD3 luminal domain, α‐helices (light blue, cyan) and β‐strands (yellow, red) of the two lobes are represented as cylinders and arrows, respectively. The HKD/HKE motifs are labeled in red text. (C) Ribbon diagram of PLD3 monomer in the crystal structure, the secondary structure colored as in panel (B). The sulfate ion bound in the catalytic site is shown in CPK style, glycosylation as white sticks and disulfide bonds as purple sticks. Electron density observed for (D) and (E) N‐linked glycosylation at N236 and N284, respectively, and (F) HKD/HKE motif catalytic site residues (2Fo‐Fc map contoured to 1.0 σ). Images in (C) – (F) were generated using the PyMOL Molecular Graphics System version 2.1.1 Schrodinger, LLC (https://pymol.org). See also Fig. 2A and Table 1.

The human phospholipase D (hPLD) enzyme family was originally characterized by its catalytic action to produce phosphatidic acid (PA) from phosphatidylcholine [17]. The family consists of six members and share a conserved HxKxxxxD (HKD for short) sequence motif which is essential for their catalytic activity [18]. The substitution of any amino acid in the motif leads to a loss of enzymatic function. However, only three members (hPLD1, hPLD2, hPLD6) have been shown to catalyze PA production. hPLD3 possesses a pair of these motifs but in its second motif the conserved aspartic acid is replaced by glutamic acid, which led to the suggestion that the enzyme might not be a functional phospholipase (Fig. 1A,B) [19]. This suggestion was consistent with the findings that there was no change in PA species in hPLD3 knockout brain lysates [14] and no demonstration of phospholipase activity [20, 21]. However, others have demonstrated hPLD3 phospholipase activity in lysosomal assays [22, 23]. Whether PLD3 has phospholipase activity remains unresolved.

hPLD3, unlike hPLD1 [24] and hPLD2 [25], has been shown to display single‐stranded 5′‐3′ exonuclease activity with an acidic pH optimum (5–5.5) and only a single copy of the HxKxxxxD motif is required [26, 27, 28]. A major physiological substrate was only recently identified with the finding that mitochondrial DNA built up in lysosomes of PLD3‐defective cells [13]. Lysosomal leakage of the mitochondrial DNA to the cytosol‐activated cGAS‐STING signaling leads to the accumulation of the C‐terminal fragment of APP, a central player in AD. STING inhibition reverses the accumulation and APP knockout in hPLD3‐deficient cells lowered STING activation. Thus, signaling between these different pathways, when dysregulated, results in neuronal endolysosomal abnormalities as observed in AD. Indeed, the toxic Aβ peptide, thought to initiate AD, arises in endolysosomes well before deposition in extracellular amyloid plaques. Interestingly, PLD3 SNP variants (M6R, K228R, N236S, N284S, T426A) all demonstrated altered exonuclease activities with SNPs clustered around the phosphodiesterase 1 domain of hPLD3 (Fig. 1A,B) showing decreased activity while those located at the termini showed increased activity [13, 29, 30].

In order to resolve some of the key controversies in the field, we have overexpressed a soluble form of the enzyme, measured its phospholipase and endonuclease activities and determined its crystal structure. We find the purified enzyme is a functional nuclease but has no phospholipase activity. We have determined the crystal structure of hPLD3 to 2.3 Å resolution which provides insights into why the enzyme does not function as a phospholipase. The structure also has been used to understand how known genetic variants associated with potential risk for AD likely impact the enzyme's structure. Our work provides a framework for developing novel therapeutics targeting hPLD3.

## Results

### Human PLD3 reveals a bilobal structure with a putative catalytic site located between the lobes

**Fig. 2:**
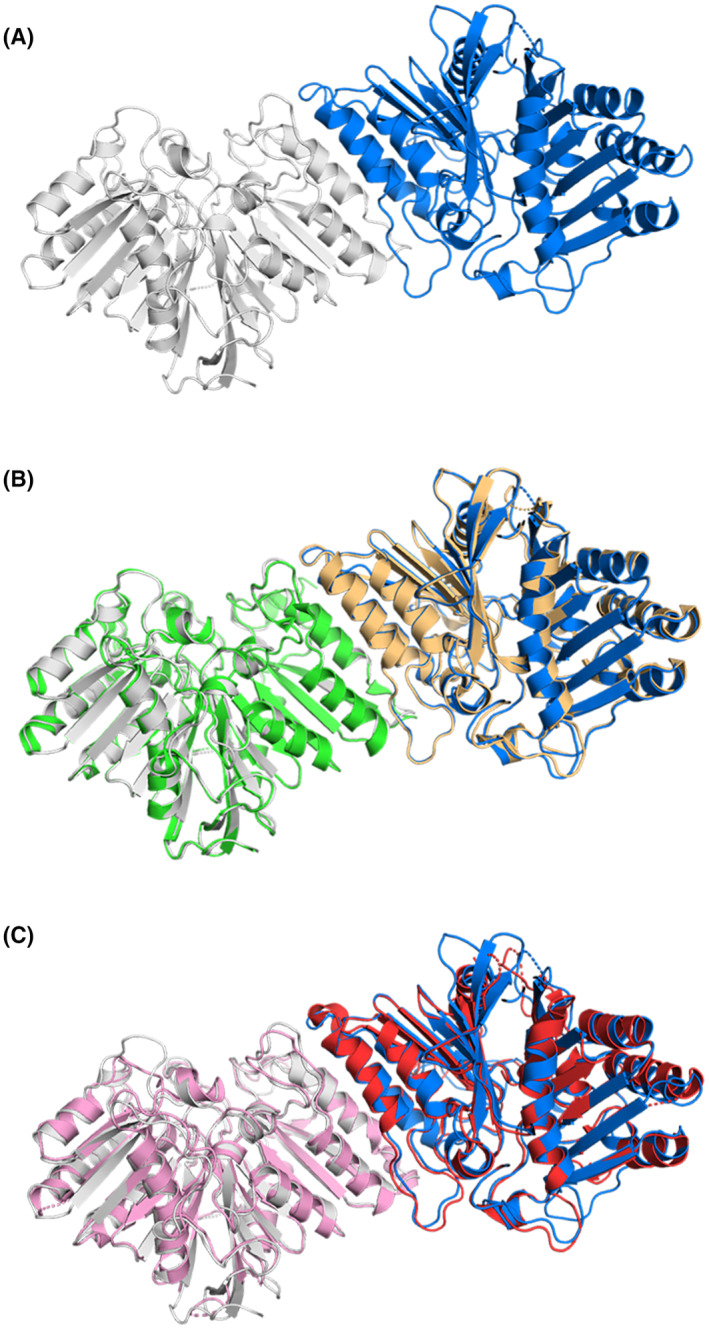
Two molecules of human PLD3 luminal domain observed in the ASU and comparison with human PLD4 ASU. (A) Two molecules of hPLD3 were observed in the ASU. Interactions at the interface between the two molecules stabilize a homodimer [29, 30]. Each molecule is shown as ribbon representation in different colors (white and blue). (B) Comparison of our structure with the hPLD3 homodimer (green and wheat colored molecules) reported by Roske *et al*. [29] (PDB: 8Q1K), the rmsd on alpha carbons of 0.43 Å. Differences between the two homodimers are observed in the conformation of the flexible loops. (C) Comparison of our hPLD3 structure with the hPLD4 homodimer (pink and red colored molecules) reported by Yuan *et al*. [30] (PDB: 8V08), the rmsd on alpha carbons of 0.79 Å. The two structures are very similar, with slight differences observed in the length and orientation of some α‐helices and β‐strands and the conformation of flexible loops. Images in (A) – (C) were generated using the PyMOL Molecular Graphics System version 2.1.1 Schrodinger, LLC (https://pymol.org). See also Table 1.

**Fig. 3:**
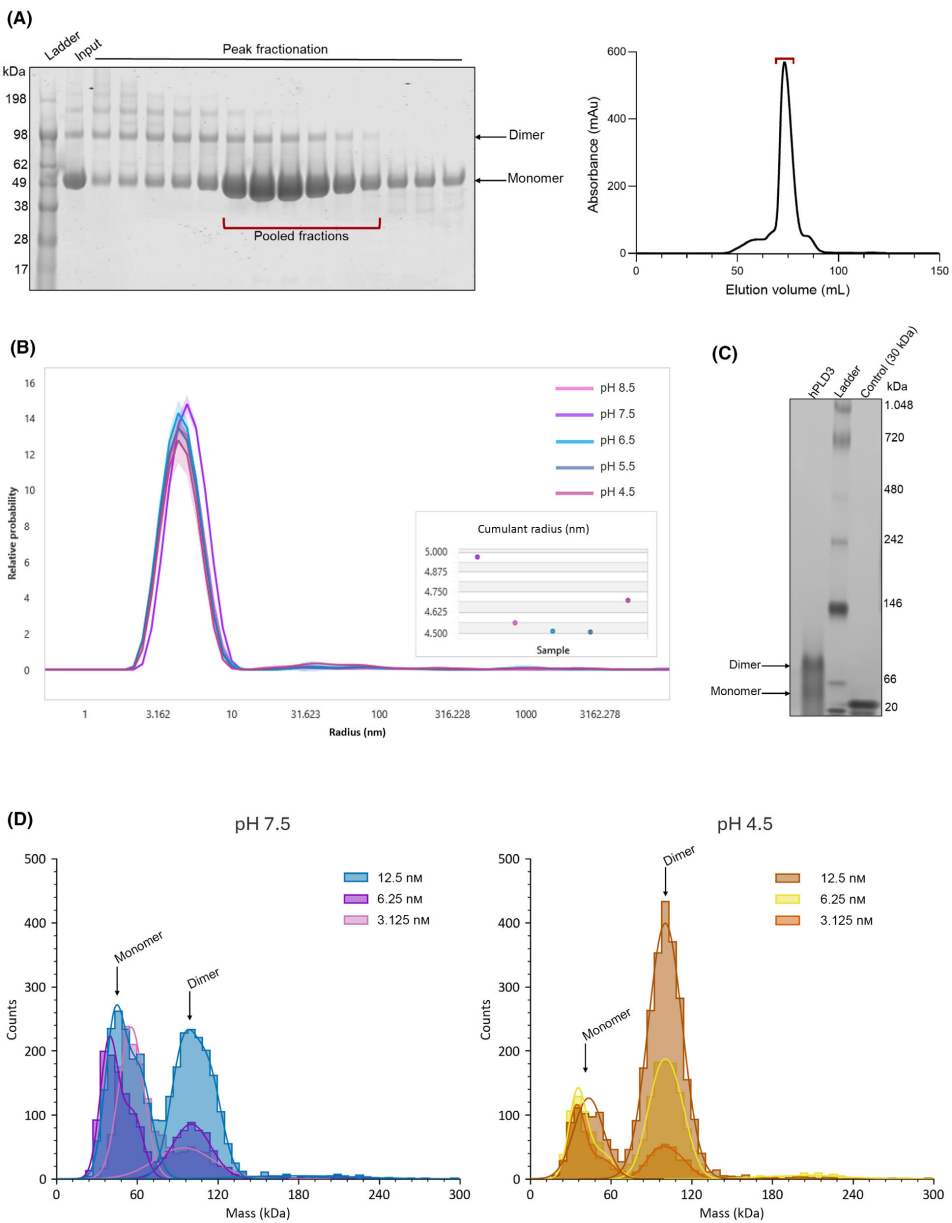
hPLD3 oligomeric state. (A) Left panel: SDS‐PAGE analysis of PLD3 size exclusion chromatography peak fractions indicating the presence of monomers and dimers, as well as possible tetramers. Right panel: size exclusion chromatography profile of PLD3 on a 16/60 Superdex 200 column (Cytiva, Marlborough, MA, USA) in 20 mm Tris pH 7.5, 250 mm NaCl (Buffer B, Table 3). Pooled fractions for the final concentration step are highlighted with a red bracket in both panels. (B) Dynamic Light Scattering (DLS) analysis of PLD3 in five different buffer conditions (Table 3) using a Prometheus Panta instrument (NanoTemper). The size distribution fits with a relative probability of detected molecule sizes for each condition are plotted against the mean hydrodynamic radius. Each sample condition was measured in triplicate and within each group 10 DLS measurements were conducted per replicate. The size distribution plots represent a sum of all the DLS scans performed, reporting the hydrodynamic radius of the averaged peak detected. (C) Analysis of PLD3 by clear native page (CN‐PAGE). The gel indicates the presence of both monomers and dimers. Results are representative of two biological repeats. (D) Histograms of mass photometry signal distribution of PLD3 at pH 7.5 (left) and 4.5 (right). Calibration was applied to all measurements while the results were plotted as mass distribution histograms. All data shown represent single experiments (but are representative of two biological repeats) and the solid lines represent the Gaussian fits.

To understand the molecular mechanisms underpinning hPLD3 function, we solved its structure by X‐ray crystallography (Figs 1C–F, 2, Table 1). The luminal domain (residues 71 to 490) was expressed in Expi293™ GnTI cells and the hPLD3 protein, following the final purification step, was analyzed by SDS‐PAGE (Fig. 3A). The gel indicated the presence of multiple species, predominantly monomers, dimers, and likely tetramers, which was consistent with the broad size exclusion chromatography peak (Fig. 3A). To characterize the observed heterogeneity and to address the protein's behavior in solution, we proceeded with further analysis. Dynamic Light Scattering (DLS) characterization was performed on a Prometheus Panta instrument (NanoTemper) by screening the purified hPLD3 in five different buffers, with pH ranging from 8.5 to 4.5 (Fig. 3B). The size distribution fits indicated a broad main peak with the main hydrodynamic radius measured at ~4.50–4.97 nm for all buffers. Even though the polydispersity measurements characterized the samples as homogeneous, the broad peak was indicative of different hPLD3 species present in solution at all pHs and all within a few nanometers of each other in size. Clear native page (CN‐PAGE) and mass photometry were then conducted to address the question of hPLD3 populations. In CN‐PAGE (Fig. 3C), two main species were identified: hPLD3 monomers and dimers. The mass photometry analysis (Fig. 3D) was in agreement and showed an equilibrium between the two species which is both pH and concentration dependent. At pH 7.5, hPLD3 monomers and dimers were observed in approximately equal proportions. Whereas at pH 4.5 ~ 77% of the protein species were dimers and ~ 21% monomers. At protein concentrations of 12.5 nm and 6.25 nm a tetramer peak was also detected, but with only 3–4% of the protein species corresponding to it. Interestingly, serial dilutions of the hPLD3 samples at both pH 7.5 and 4.5 revealed that at lower concentrations (3.125 nm) hPLD3 exhibits a preferred monomeric behavior.

**Table 1:** Crystallographic data and refinement statistics.

Data collection	
Space group	*C2*
Cell dimensions	
*a*, *b*, *c* (Å)	205.1, 60.6, 111.5
β (°)	98.7
Wavelength (Å)	0.956
Resolution (Å)	49.0–2.30 (2.36–2.30)^a^
No. of observations	206 144 (15061)
No. of unique reflections	60 087 (4455)
*R*_meas_ (%)	7.9 (85.1)
*R*_pim_ (%)	4.2 (45.8)
CC_1/2_	0.997 (0.683)
*I*/σ_*I*_	10.2 (1.6)
Completeness (%)	99.3 (99.6)
Multiplicity	3.4 (3.4)
Wilson B‐factor (Å^2^)	46.4
Refinement	
*R*_work_/*R*_free_ (%)	18.5 (33.5)/20.9 (34.2)
No. of non‐hydrogen atoms	
Protein	6530
Ligands	180
Solvent	387
Average B‐factors (Å^2^)	
Protein	58.0
Ligands	84.5
Solvent	56.5
R.m.s. deviations from ideal geometry	
Bond lengths (Å)	0.01
Bond angles (°)	1.15
Ramachandran plot	
Favored (%)	96.1
Allowed (%)	3.9

^a^ Statistics for the highest resolution shell are shown in parentheses.

Crystals were obtained that diffracted to 2.3 Å resolution and the structure was determined by molecular replacement using an AlphaFold2 model in 2022 (AF‐Q8IV08‐F1‐model_v4.pdb). The crystal structure revealed four of the eight cysteines formed two pairs of disulfide bonds, namely C81‐C237 and C366‐C487 (Fig. 1A,C). In our structure, cysteine residues C77 and C239 are close to each other but are not orientated so as to form a disulfide bond (as indicated by a dashed line in Fig. 1A); however, the disulfide bond is observed in the crystal structure obtained by Roske *et al*. [29]. We cannot exclude they do form a disulfide in solution and that the synchrotron X‐ray beam has broken the bond. C300 is completely buried whereas C282 is surface exposed and appears to be interacting with a chain of water molecules. Previously, two N‐glycosylation sites at asparagine residues 97 and 132 that begin with N‐Acetylglucosamine (GlcNAc) were identified [31]. Three more N‐glycosylation sites at asparagine residues 236, 284 and 387 have been experimentally verified [29, 32]. The presence of two N‐linked oligosaccharide (GlcNAc) modifications at residues N236 and N284 (Fig. 1A,D,E) were clearly observed in the electron density map. N97 is located on a mobile loop so whether it is modified remains uncertain; there is no evidence of N‐glycosylation for N132 and we observed some trailing density off N387 but not definitive enough to build in carbohydrate. There was no evidence of metal ions in the anomalous difference Fourier maps. Two hPLD3 molecules were found packed in the asymmetric unit (ASU, Fig. 2A) and found to closely superimpose (rmsd on alpha carbons of 0.12 Å). The final structure superimposed closely with the AlphaFold2 model (rmsd on alpha carbons of 0.39 Å).

The hPLD3 homodimer we observed in the ASU was recently reported by Roske *et al*. [29] and a similar homodimer reported for hPLD4 [30]. A comparison of the homodimers revealed they were very similar, with rmsd on alpha carbons of 0.43 and 0.79 Å respectively, when compared to our structure (Fig. 2B,C). Differences in the conformation adopted by flexible loops was observed between the two hPLD3 homodimers, whereas there were also slight differences in the length and orientation of some α‐helices and β‐strands when comparing our hPLD3 homodimer with that of hPLD4. A detailed description of the monomer‐monomer interface in the hPLD3 and hPLD4 homodimers, along with the stability and functional effect of key residues at this interface, has recently been reported by Roske *et al*. [29] and Yuan *et al*. [30].

The crystal structure reveals that the luminal domain of hPLD3 is shaped like a horse saddle, composed of two lobes with each lobe containing one HKD/HKE sequence motif. There are seven core β‐strands that are interconnected by α‐helices to form an overall α‐β two‐layered sandwich topology (Fig. 1B,C), which is reminiscent of the published structures of hPLD1/2 [33, 34] and orthologous PLD enzymes [35, 36, 37]. The nomenclature adopted here for the secondary structure features within the hPLD3 protein fold has been made consistent with those published for the hPLD1 and hPLD2 crystal structures [34]. The N‐terminal lobe has seven β‐strands that are all under seven residues long and eight α‐helices of varying length from 4 to 18 residues. The C‐terminal lobe has eight β‐strands, however, β8 and β9 may form one long β‐strand depending upon conformational flexibility. Similarly, there are eight α‐helices of varying length in the C‐terminal lobe. Together, the two lobes form a core structure composed of two β‐sheets, each with two pairs of interchanging parallel and antiparallel β‐strands and three parallel β‐strands. Notably, there is at least one α‐helix present in between each β‐strand, except for β‐strands that contain the HKD/HKE motif (β5 and β13).

**Fig. 4:**
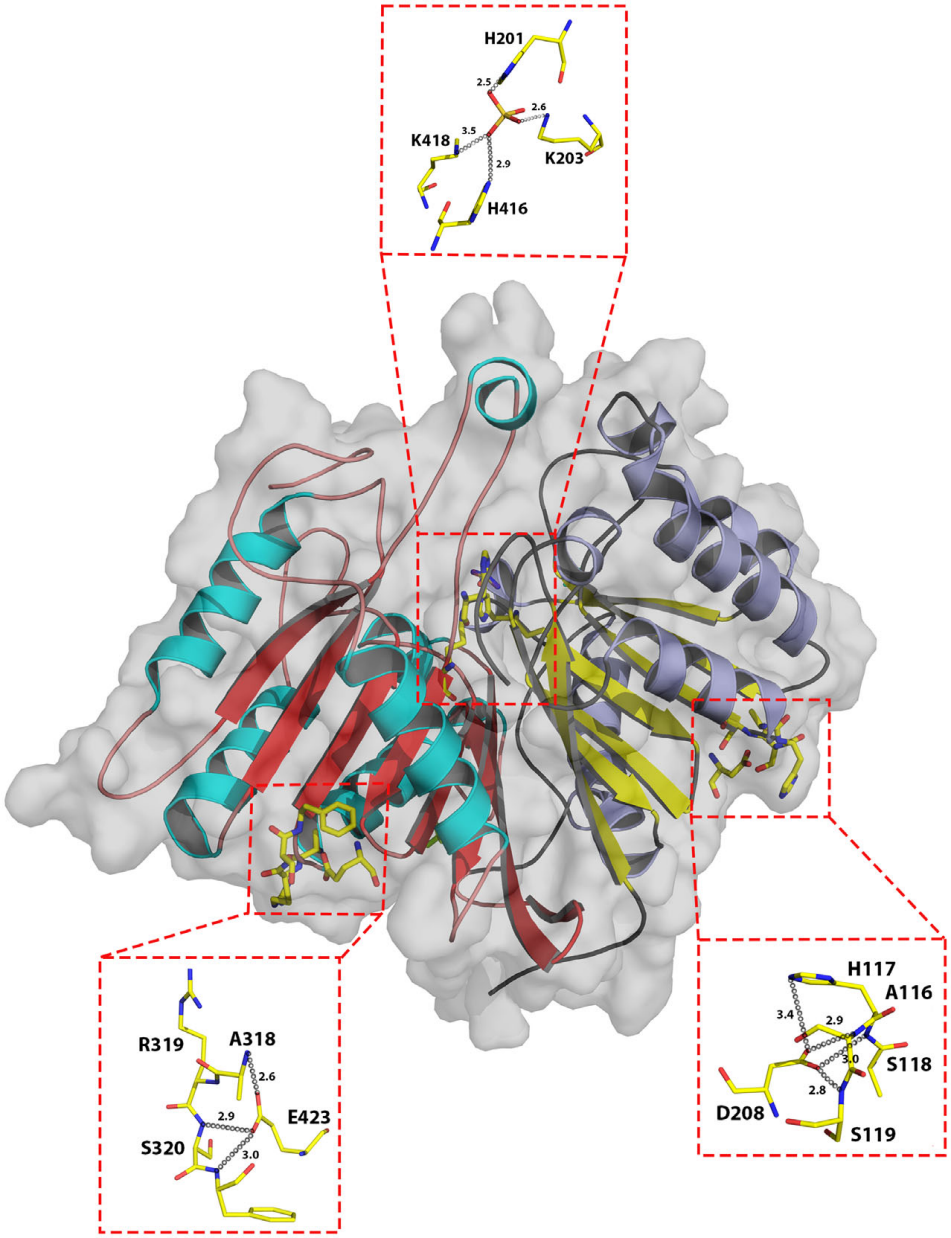
Location of HKD/HKE catalytic motifs in the hPLD3 crystal structure. Residues H201, K203, H416 and K418 come together to form the catalytic site at the bottom of a funnel‐shaped cavity located at the domain interface. These residues are involved in a network of polar interactions with a sulfate ion (top zoom box). Residues D208 and E423 are distal from the catalytic site (bottom zoom boxes). Gray dashed lines indicate possible hydrogen bonding interactions with their corresponding distances shown in Å. Images were generated using the PyMOL Molecular Graphics System version 2.1.1 Schrodinger, LLC (https://pymol.org). See also Fig. 6.

The catalytic pair of histidine and lysine residues of the HKD/HKE motifs (H201, K203, H416 and K418; Fig. 1F) come together at the bottom of a funnel‐like cavity to form the putative catalytic site (Fig. 4). A sulfate ion from the crystallization conditions (Figs 1C, 4), identified in the electron density map, was found to form hydrogen bonding interactions with these histidine and lysine residues. Consistent with this observation is the finding that vanadate, a sulfate mimic, inhibits PLD3 activity (with an IC50 of 10 μm) which could be reversed by EDTA [28]. The hPLD3 putative catalytic site location, at the bottom of the funnel‐shaped cavity between the lobes, is consistent with the location observed in the hPLD1, hPLD2 and hPLD4 structures. Like hPLD1 and PLD2, residues D208 and E423 of the sequence motifs were found to be distal from the catalytic site, located on opposing faces of the protein (Fig. 4).

### Mapping of risk variants reveals most are distal from the catalytic site

**Fig. 5:**
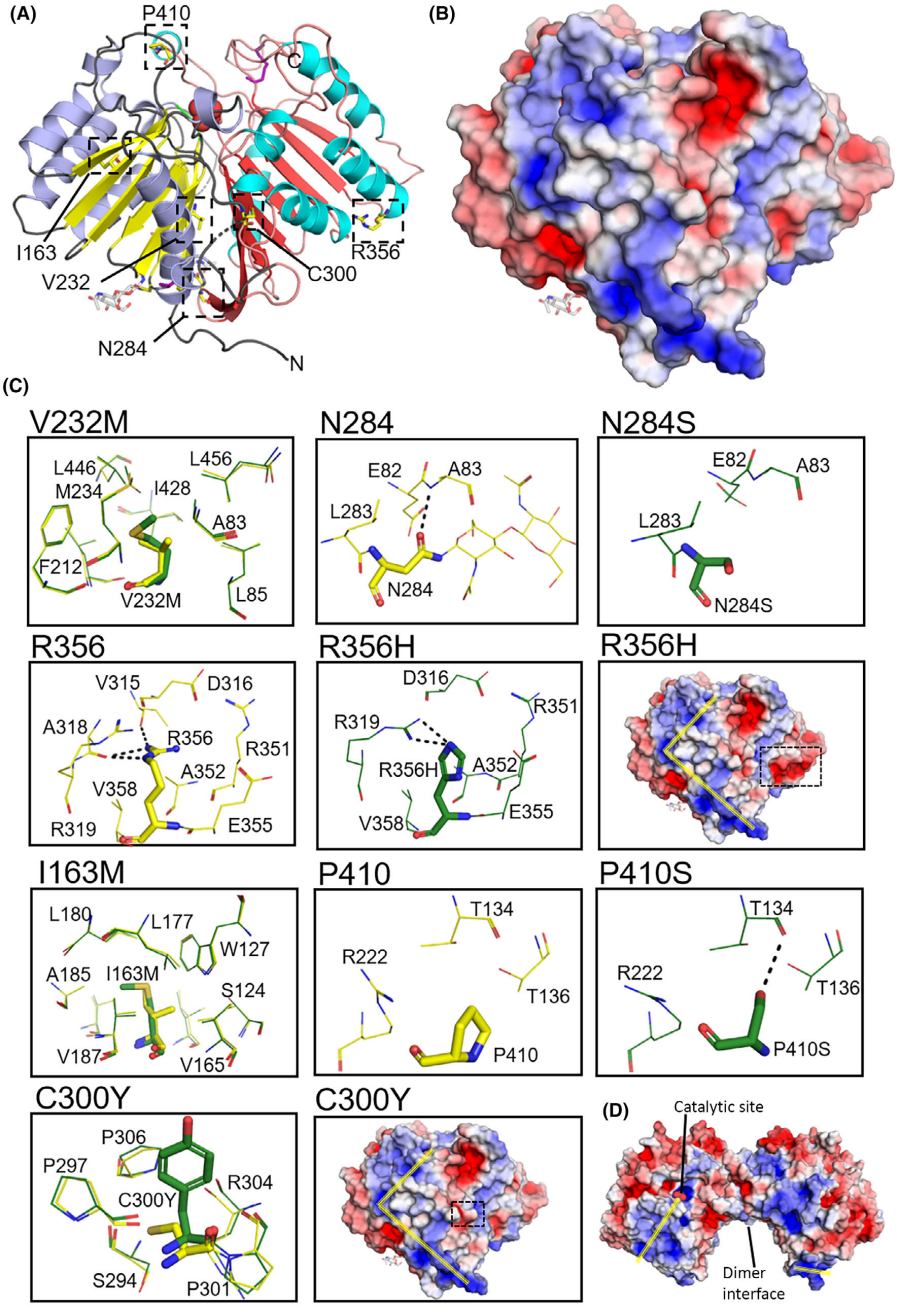
Mapping of AD risk variants onto the hPLD3 structure. (A) The location of six AD risk variants is indicated by dashed black boxes and (B) the electrostatic surface potential for the same view as panel (A) is shown. (C) The immediate environment surrounding the native and variant residues are shown in the zoomed‐in views. Each point mutation variant was modeled *in silico* and compared to the crystal structure. Residues from the crystal structure have yellow colored bonds and those from the variant model are colored dark green. The variant residue is shown in stick format. For N284, the N‐linked GlcNAc is shown as thin sticks, and removal of the glycosylation may affect PLD3 stability and/or activity. Black dashed lines indicate possible hydrogen bonding interactions for the side‐chains of N284, R356, R356H and P410S. Where there was a change in hydrogen bond interaction patterns, between the native or variant side‐chain and surrounding residues, the native and variant structures are shown individually. For I163M, V232M and C300Y the native and variant structures are shown as overlays. The I163M, V232M, N284S, C300Y and R356H variants were predicted to be destabilizing using DDMut, ie. they all have ΔΔG^stability wt>mt^ < 0 [39]. Accommodation of the larger side‐chains is a major issue for the I163M, V232M and C300Y variants. There was no change in the electrostatic surface potential for the I163M, V232M, N284S and P410S variants (not shown) compared to wild‐type (panel B). The electrostatic surface potential for the C300Y and R356H variants (location indicated by the black dash boxes) differed to that of the wild‐type; however the residues are located well away from the putative binding route of ssDNA suggested by Roske *et al*. [29] (yellow lines) and unlikely to affect ssDNA binding. (D) The electrostatic surface potential for the hPLD3 homodimer showing the putative binding route of ssDNA suggested by Roske *et al*. [29]. The sulfate ion observed in the catalytic site of our structure is shown as CPK. Images in (A) – (D) were generated using the PyMOL Molecular Graphics System version 2.1.1 Schrodinger, LLC (https://pymol.org). The PyMOL APBS Electrostatics Plugin tool was used to calculate the electrostatic potential surface shown in panels (B) – (D).

Genome‐wide association studies and Next‐generation sequencing studies have identified several point mutations and synonymous mutations in hPLD3 DNA or RNA. AD risk variants resulting from the mutations include I163M, V232M, N284S, C300Y, R356H and P410S, however only V232M has a clear effect (2‐fold increase) on AD risk [1, 6, 7, 9, 22, 38]. The availability of the crystal structure of hPLD3 provides a framework for understanding the effect of AD risk variants associated with the enzyme. Therefore, *in silico* mutagenesis was performed using the crystal structure of hPLD3 to spatially map six variants that are present in the luminal domain (I163M, V232M, N284S, C300Y, R356H and P410S) (Fig. 5A–C).

Notably, all of the residues are distal from the catalytic site. Residues I163 and V232 are located in hydrophobic pockets and make numerous van der Waals contacts with the surrounding residues. V232 is located at the interface between the N‐terminal and C‐terminal lobes of PLD3, while I163 is located on a β‐strand with its side‐chain poking up between two α‐helices. The larger methionine side‐chain can be accommodated in both the I163M and V232M variants; however, the side‐chain conformation is restricted and conformations deviating from those shown in Fig. 5C result in steric contact with surrounding residues. From our *in silico* modeling it is likely that the I163M and V232M variants are destabilizing, potentially reducing the level of folded PLD3 expressed. It is unknown whether C300 plays any role in protein–protein interactions and the larger tyrosine side‐chain of C300Y can only be accommodated by rotating away from the interior of the protein to become solvent exposed. The N284S variant knocks out the N‐linked glycosylation site (Fig. 1A,E) and may have an effect on PLD3 expression levels, transport or function. R356 is located on the solvent‐exposed end of an α‐helix, at the monomer‐monomer interface of the homodimer, with the side‐chain poking back toward the interior of the C‐terminal lobe of PLD3. The histidine side‐chain of the R356H variant is easily accommodated. P410 is located on a flexible loop and the effect of the P410S variant on PLD3 stability is unknown.

Recently Roske *et al*. [29] examined the electrostatic surface potential of hPLD3 and proposed the binding route for ssDNA (Fig. 5D). We compared the effect of the AD risk variants on the electrostatic surface potential of hPLD3 (Fig. 5A–C) and found that there was no change in the electrostatic surface potential for the I163M, V232M, N284S and P410S variants (not shown) compared to wild‐type. The electrostatic surface potential for the C300Y and R356H variants differed from that of the wild‐type; however, the residues are located well away from the putative binding route of ssDNA (yellow lines in R356H and C300Y panels, Fig. 5C) and unlikely to affect ssDNA binding.

The effect on protein stability for each variant was also predicted using DDMut [39]. I163M, V232M, N284S, C300Y and R356H were all predicted to be destabilizing (Table 2). In contrast, P410S was predicted to be stabilizing. Replacement of the proline with a serine at position 410 introduces the ability to hydrogen bond with nearby residues (Fig. 5C) and also increases backbone flexibility at this location. The predictions for I163M and V232M are consistent with the decreased thermal melt temperature (*T*_m_) of these variants reported recently by Yuan *et al*. [30].

**Table 2:** Predicted stability of hPLD3 AD risk variants. The effect of each amino acid substitution upon protein stability was predicted using DDMut (Zhou *et al*. [39]). A ΔΔG^stability wt>mt^ < 0 indicates a destabilizing mutation whereas ΔΔG^stability wt>mt^ ≥ 0 indicates a stabilizing mutation.

AD risk variant	ΔΔG^stability wt>mt^ (kcal⋅mol^−1^)
I163M	−1.09
V232M	−0.59
N284S	−0.15
C300Y	−2.63
R356H	−0.33
P410S	+0.24

Recently, site‐directed mutagenesis studies were conducted to explore the effect of some of these variants on expression levels, proteolytic processing, intracellular transport and 5′‐3′ exonuclease activity [28, 30]. The N‐terminal M6R AD risk variant was found to show no difference compared to wild‐type [28]. The I163M variant lost 5′‐3′ exonuclease activity [30]. Cappel *et al*. [28] found that the V232M variant exhibited reduced 5′‐3′ exonuclease activity but the variant was expressed slightly lower than wild‐type enzyme. This implied that the 5′‐3′ exonuclease activity was only mildly reduced or similar to the wild‐type and that the point mutation may be slightly destabilizing as discussed above. In contrast, Yuan *et al*. [30] found that the V232M variant had a slightly increased 5′‐3′ exonuclease activity.

### Key structural features of other human PLDs missing in hPLD3

**Fig. 6:**
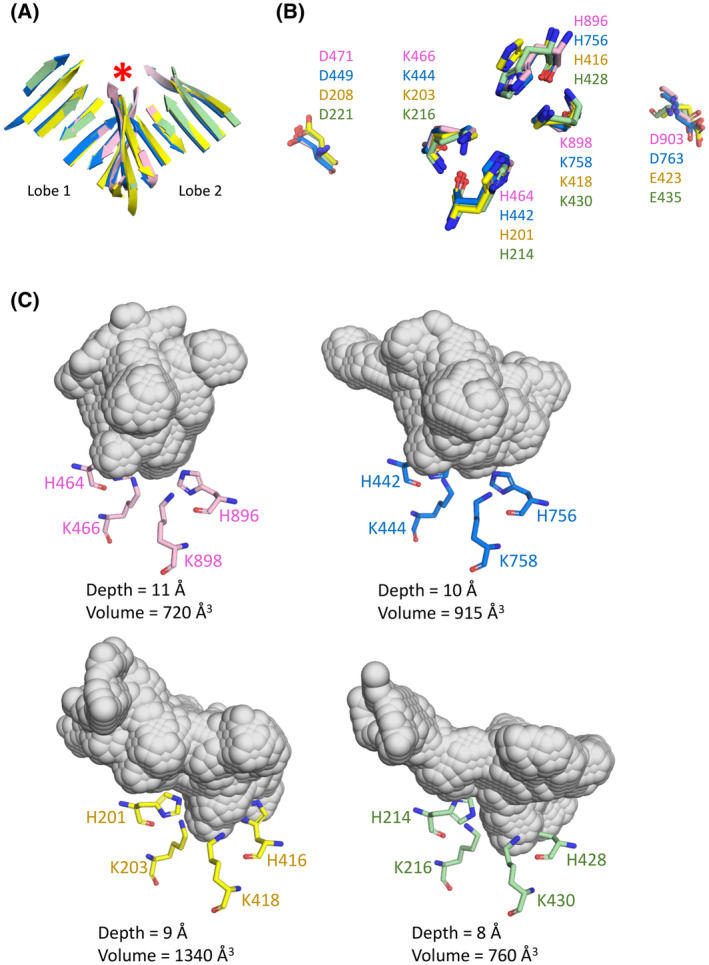
Conserved features in the human PLD1, PLD2, PLD3 and PLD4 structures and differences in the solvent‐accessible catalytic cavity. (A) Superposition via secondary structure elements of PLD1 (pink, PDB: 6U8Z), PLD2 (blue, PDB: 6OHP), PLD3 (yellow) and PLD4 (green, PDB: 8V08) reveals the catalytic core is conserved, shown are the two central β‐sheets of the α‐β two‐layered sandwich (also known as horse saddle topology). For clarity, the conserved α‐helices of the α‐β two‐layered sandwich are not shown. (B) Location of the catalytic HKD/HKE motifs of PLD1 (pink), PLD2 (blue), PLD3 (yellow) and PLD4 (green). (C) The solvent‐accessible funnel (gray surface) that leads to the catalytic site is shown for PLD1 (pink), PLD2 (blue), PLD3 (yellow) and PLD4 (green). The depth and volume of each solvent‐accessible funnel are listed. Images in (A) – (C) were generated using the PyMOL Molecular Graphics System version 2.1.1 Schrodinger, LLC (https://pymol.org). The alignment shown in (A) was generated using MUSTANG version 3.2.3 and the solvent‐accessible surfaces shown in (C) were calculated using KVFinder‐web (as described in Methods).

Recently, the crystal structures of hPLD1 (PDB: 6U8Z, 6OHR) [33, 34], hPLD2 (PDB: 6OHM, 6OHO, 6OHP, 6OHQ, 6OHS) [34] and hPLD4 (PDB: 8V08) [30] catalytic domains were determined. Although there is conservation of secondary structure features in their catalytic cores (Fig. 6A), low pairwise sequence identity has limited the understanding of human PLD enzymes. Indeed, the pairwise sequence identity between hPLD3 and hPLD1 or hPLD2 is < 14%. Like hPLD3, the bilobed architecture of hPLD1, hPLD2 and hPLD4 allows each copy of the HKD catalytic motif (or HKD/HKE motif in the case of hPLD4) to converge and form the funnel‐like catalytic pocket [30, 33, 34]. The spatial locations of the catalytic histidine and lysine residues are also highly conserved in hPLD3 and hPLD4. Notably, the aspartic acid of the HKD motifs in hPLD1 and hPLD2, and glutamic acid in the HKE motif of hPLD3 and hPLD4, are spatially conserved (Fig. 6B). The solvent accessible volume of the hPLD1, hPLD2 and hPLD4 catalytic pocket is smaller than that observed for hPLD3 (Fig. 6C).

**Fig. 7:**
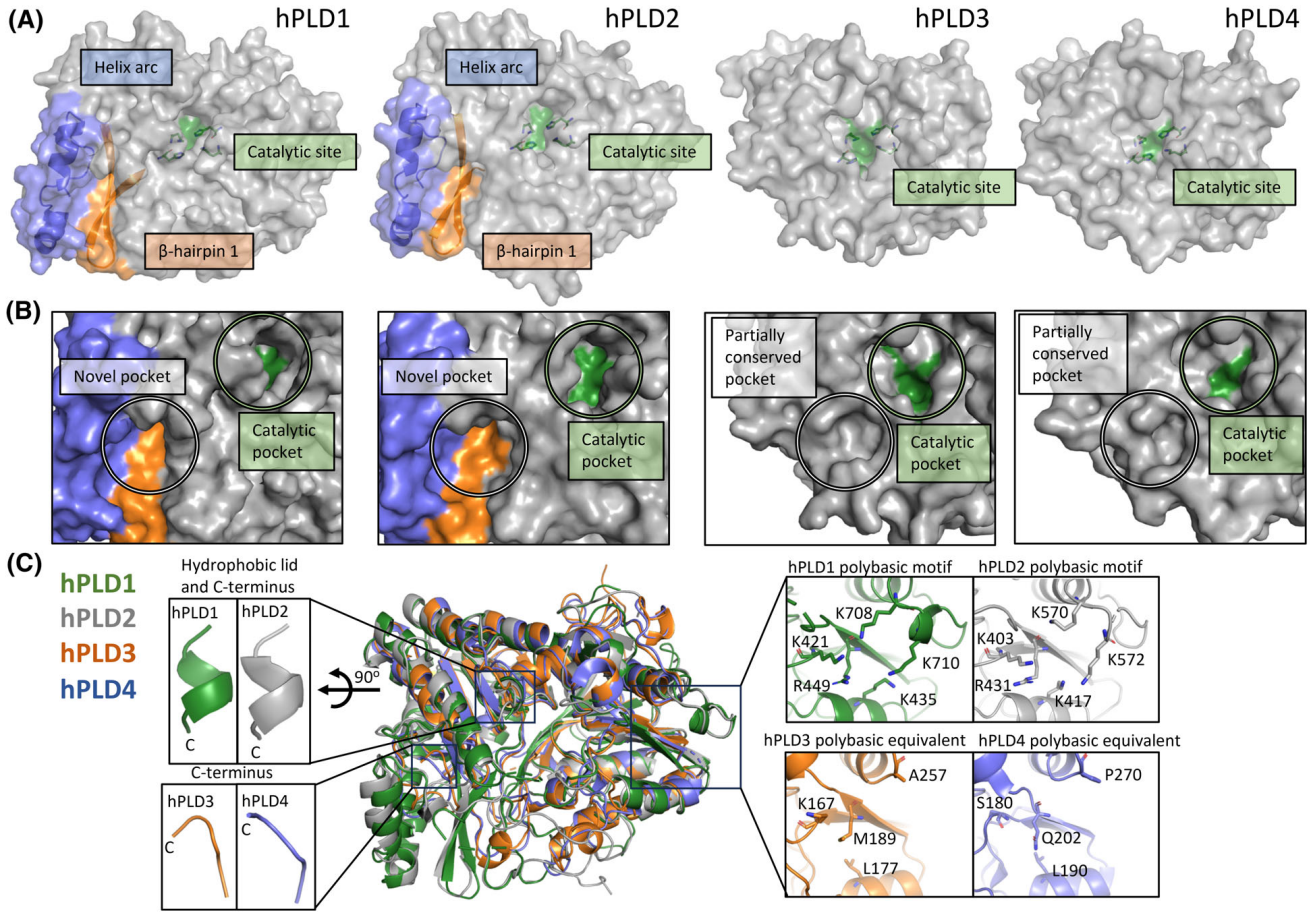
Differences in the human PLD1, PLD2, PLD3 and PLD4 structures. In PLD1 and PLD2 a helix arc and β‐hairpin form a novel pocket that is located adjacent to the catalytic pocket [34] but these two features are not present in either PLD3 or PLD4 and this pocket is only partially conserved. The novel pocket in PLD1 and PLD2 has been proposed to be an allosteric pocket [34]. (A) Crystal structure of PLD1 (PDB: 6U8Z), PLD2 (PDB: 6OHP), PLD3, and PLD4 (PDB: 8V08). The catalytic site (green), β‐hairpin 1 (orange) and helix arc (blue) are shown. (B) The solvent accessibility of the catalytic pocket and novel pocket in PLD1, PLD2, PLD3, and PLD4 are highlighted. (C) Key residues in PLD1 and PLD2 that are absent in PLD3 and PLD4. The zoom boxes show a polybasic motif and a C‐terminal 3_10_ helix that is not conserved in PLD3 or PLD4. Images in (A) – (C) were generated using the PyMOL Molecular Graphics System version 2.1.1 Schrodinger, LLC (https://pymol.org). See also Fig. 6.

The N‐terminal lobe of the hPLD3 (and hPLD4) luminal domain resembles that of hPLD1 and hPLD2. In contrast, the C‐terminal lobe differs significantly with many structural features missing. In this lobe, both hPLD1 and hPLD2 have a region comprised of α‐helices and long flexible loops of approximately 30 residues that has been termed the ‘helix arc’ (hPLD1 residues 966–999; hPLD2 residues 861–896) (Fig. 7A). The helix arc wraps around the C‐terminal lobe, and together with a β‐hairpin, forms a so‐called “novel” pocket (volumes of 842 Å^3^ and 1162 Å^3^, respectively) adjacent to the catalytic pocket (Fig. 7B) [34]. Based on the inter‐connection of residues forming the novel pocket with catalytic site residues, Metrick *et al*. [34] proposed that this pocket may be an allosteric site in hPLD2 and a target for developing allosteric inhibitors. This novel pocket is only partially conserved in hPLD3 and hPLD4 due to the absence of β‐hairpin 1 and the helix arc, which flank the edges of the pocket in hPLD1 and hPLD2 (Fig. 7A,B).

Another distinguishing feature is the presence of a so‐called hydrophobic ‘lid’ in hPLD1 and hPLD2 (Fig. 7C) which is not present in either hPLD3 or hPLD4. The hydrophobic ‘lid’ is a distinct feature from the helix arc described above. In hPLD1, C‐terminal threonine residues form hydrogen bonding interactions with residues near the catalytic site [33]. In both enzymes the C‐terminal 3_10_‐helix forms a hydrophobic ‘lid’, which directs itself toward the lower hydrophobic section of the funnel‐like catalytic site. In the hPLD1 crystal structure, both the C‐terminal 3_10_‐helix and the threonine residues stabilize the catalytic site conformation. It has been postulated that this hydrophobic ‘lid’ interacts with the tail of lipid substrates, such as PtdCHO, and positions them properly for hydrolysis [34]. Mutagenesis studies have shown that mutations of the C‐terminal 3_10_‐helix or the C‐terminal threonine residues cause hPLD1/2 to be enzymatically inactive [40, 41]. The spatial locations of the hydrophobic ‘lid’ of hPLD1/2 are conserved (Fig. 7C). The C‐terminal 3_10_‐helix is absent in the hPLD3 crystal structure and the C‐terminal region contains C487, which is involved in a disulfide bond with C366, making a conformational conversion of the C‐terminal region into a 3_10_‐helix unlikely. The C‐terminal 3_10_‐helix is also absent in the hPLD4 crystal structure. The C‐terminal hPLD3 residue is a leucine (L490) which cannot mimic the same interactions as the C‐terminal threonine residue in hPLD1/2.

Finally, hPLD1 requires phosphatidylinositol 4,5‐bisphosphate (PIP2) as a co‐factor for both basal activity and activation by RhoA and other GTPases [33]. The polybasic motif in hPLD1 accommodates the binding of PIP2 (Fig. 7C) and this motif is also present in hPLD2. In contrast, neither hPLD3 nor hPLD4 have this polybasic motif and neither have been reported to interact with, or be activated by, GTPases.

### PLD3 is a nuclease

**Fig. 8:**
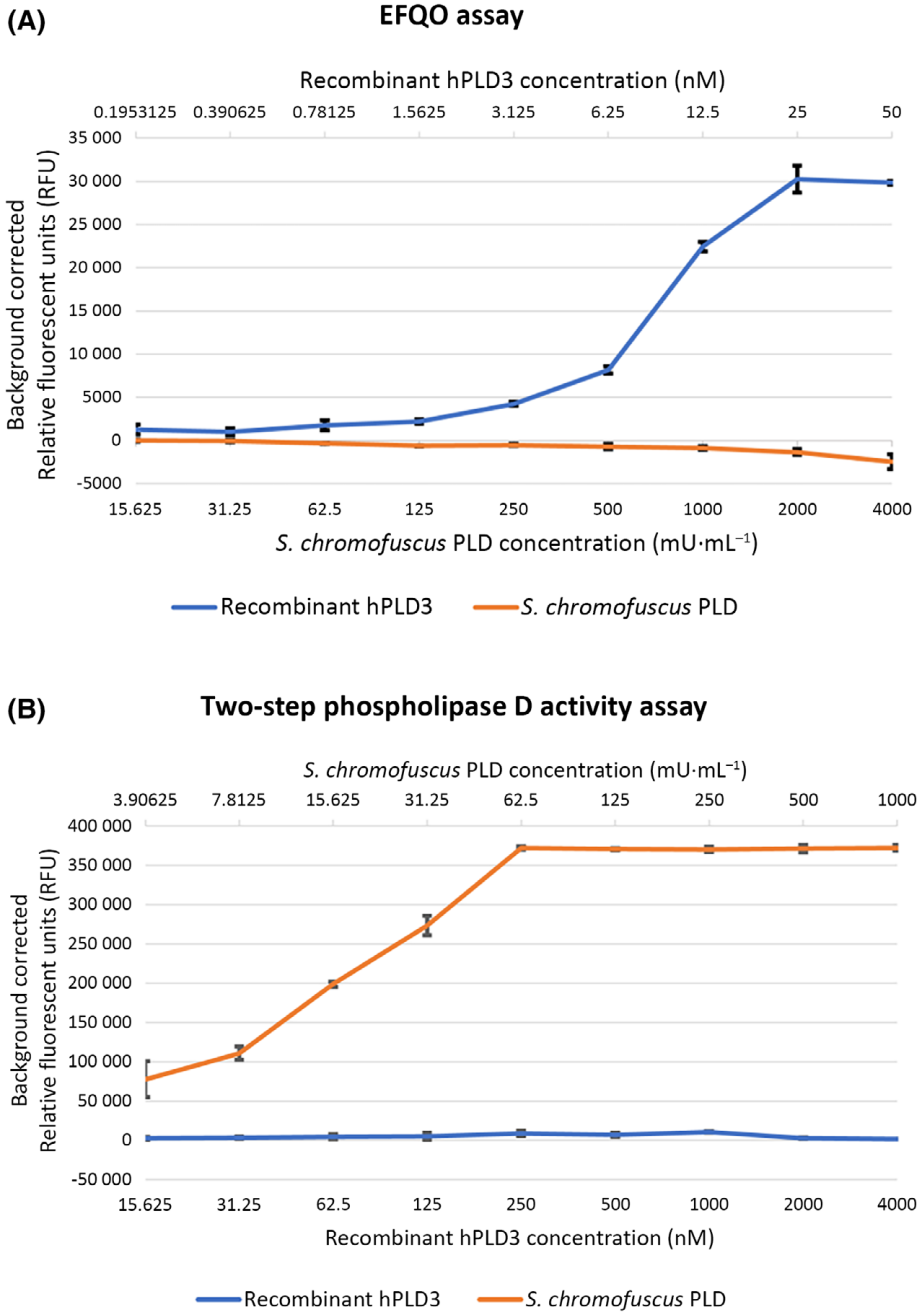
Testing recombinant hPLD3 for 5′ exonuclease and phospholipase D activity. (A) The 5′ exonuclease activity was tested by the end‐labeled fluorescence‐quenched oligonucleotide (EFQO) assay. The graph displays released fluorescence against recombinant hPLD3 concentration following incubation for 2 h at 37 °C with 1 pmol⋅μL^−1^ of FAM‐ssDNA‐substrate in a MES pH 5.5 containing buffer. A negative control PLD from *S. chromofuscus* was included. Data are shown as the mean ± SD of *n* = 3 independent experiments performed in triplicate. (B) The phospholipase D activity was tested by the Amplex® Red Phospholipase D Assay Kit (ThermoFisher Scientific) using the two‐step assay method. The graph displays released fluorescence against PLD concentration following incubation for 2.5 h at 37 °C with 100 mm lecithin solution substrate in a MES pH 5.0 containing buffer. A positive control PLD from *S. chromofuscus* was included. Data are shown as the mean ± SD of *n* = 4 independent experiments performed in duplicate.

Our purified enzyme was examined for 5′ exonuclease activity [28]. The recently reported EFQO assay [28] was performed under the acidic conditions associated with the lysosomal environment where PLD3 is trafficked [14]. hPLD3 exhibited acidic 5′ exonuclease activity; cleaving the 5′ end of the FAM‐ssDNA‐substrate under acidic conditions to release the non‐quenched fluorophore coupled nucleotide, measured by an increase in FAM fluorescence (Fig. 8A). This activity was hPLD3 concentration‐dependent and measurable at low concentrations. Under the conditions studied, incubating hPLD3 with 1 pmol⋅μL^−1^ fluorescent substrate for 2 h at 37 °C, maximal fluorescence was observed at 25 nm hPLD3. It has been shown that hPLD3 has the highest exonuclease activity for a ssDNA oligomer with a thymidine in the 5′ position [29]. The mechanism of action has also been characterized confirming the importance of the two catalytic site histidines, as when either or both are mutated to alanine the 5′ exonuclease activity of PLD3 is completely abolished [30].

### PLD3 is not a phospholipase

The phospholipase D reaction is a two‐step process. First, the catalytic histidine residue (of the catalytic HKD motif) acts as a nucleophile that attacks the phosphate group of the substrate to form a phosphoenzyme intermediate. The histidine from the other catalytic HKD motif extracts protons from a water molecule, allowing the activated water molecule to hydrolyze the phosphoenzyme intermediate to yield metabolites [42].

Having established the purified enzyme has 5′ exonuclease activity we then examined whether it has phospholipase activity. The assay was performed under the acidic conditions associated with the lysosomal environment where PLD3 is trafficked [14]. hPLD3 did not display any phospholipase activity under neutral or acidic conditions, at concentrations up to 8 μm, when compared to the *S. chromofuscus* PLD positive control (Fig. 8B).

**Fig. 9:**
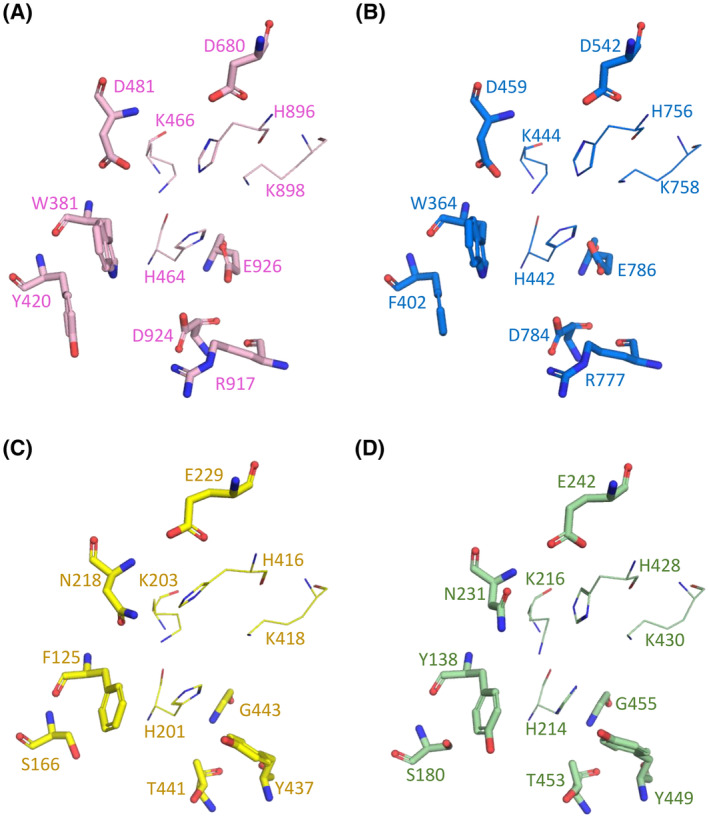
Differences in the catalytic sites of human PLD1, PLD2, PLD3 and hPLD4. Residue differences observed in the catalytic site for (A) PLD1, (B) PLD2, (C) PLD3 and (D) PLD4. PLD1 and PLD2 residues involved in conserved interaction networks are shown. Superposition reveals that four residues in PLD3 (T441, G443, N218, E229) and PLD4 (T453, G455, N231, E242) are not conserved. E926 in PLD1 and E786 in PLD2 have been identified as the essential acidic residue involved in the phospholipase mechanism [34]. Both PLD3 and PLD4 have a glycine residue in this position (G443 and G455, respectively). E229 of PLD3 is proposed to be involved in the 5′ exonuclease cleavage mechanism [29], and this residue is conserved in PLD4 (E242). Both PLD1 and PLD2 have an aspartic acid residue (D680 and D542, respectively) ~2 Å away from the location of E229 (or E242 in PLD4). Residues involved in lipid substrate recognition and catalysis for PLD1 (W381, Y420, R917) and PLD2 (W364, F402, R777) are not conserved in PLD3 (F125, S166, Y437) or PLD4 (Y138, S180, Y449). Images in (A) – (D) were generated using the PyMOL Molecular Graphics System version 2.1.1 Schrodinger, LLC (https://pymol.org).

We noted in the above structural comparisons a number of key differences between hPLD1/2 and hPLD3 including low sequence identities, an aberrant HKE motif, the absence of a ‘hydrophobic lid’ and a much wider catalytic pocket. Previously, the helix arc at the C‐terminus was shown to be required for activity in hPLD1/2 [34] but we find it is completely absent in both hPLD3 and hPLD4 crystal structures. In hPLD1/2 there are conserved networks of interactions surrounding the catalytic site but a superposition of crystal structures reveals that three equivalent residues in hPLD3 (T441, G443, N218) are not conserved (Fig. 9). In particular, the replacement of glutamic acid by glycine at position 443 in hPLD3 (G455 in hPLD4; Fig. 9C,D) means that G443 (or hPLD4 G455) cannot generate the required nucleophilic histidine residue in the catalytic site that E926 in hPLD1 or E786 in hPLD2 can for the phospholipase reaction to occur. Instead, Roske *et al*. [29] have proposed that E229 in hPLD3 is the acidic residue required for the 5′‐exonuclease reaction (the equivalent residue in hPLD4 is E242). The crystal structures also reveal that residues in hPLD1 (W381, Y420, R917) and hPLD2 (W364, F402, R777) responsible for lipid substrate recognition and catalysis are not conserved in hPLD3 (F125, S166, Y437) or hPLD4 (Y138, S180, Y449) (Fig. 9). Finally, the PIP2 binding site in the polybasic pocket is not conserved in hPLD3 and indeed two lysine residues are completely absent (Fig. 7C). These differences, together with contradictory reports in the literature raised questions about whether the enzyme was a true phospholipase.

## Discussion

hPLD3 polymorphisms have been linked to late‐onset AD over the last decade. More recently, a direct link between PLD3 and the disease has been revealed [12, 13]. However, informed development of hPLD3 modulators has been hindered by a lack of structural knowledge. We report here the crystal structure of the luminal domain of hPLD3 to 2.3 Å resolution (Figs 1C, 2A, 4, Table 1) which reveals a bilobal structure with the catalytic site located in a funnel‐shaped crevice between the two lobes (Fig. 4). A sulfate ion was identified in the site, binding to some of the key residues of the HKD sequence motif (Figs 1C, 4).

There are small differences between the luminal domain of the hPLD3 AlphaFold2 model (AF‐Q8IV08‐F1‐model_v4.pdb) and our experimental structure (rmsd on alpha carbons of 0.39 Å). There are differences in the backbone conformation of flexible loops and the conformation of some unconstrained side chains (for example S169, E172, T202, M327, F335 and H337). In the catalytic site, with the exception of residue H416 (of the HKE motif), there is generally good agreement in side‐chain conformations between the AlphaFold2 model and our hPLD3 experimental structure (<0.5 Å difference between side‐chain atoms). The side‐chain of H416 in the AlphaFold2 model would sterically clash with both the bound sulfate ion that we observe in our crystal structure and the ssDNA observed in the complex reported by Roske *et al*. [29] (PDB: 8Q1K). We considered all available side‐chain rotamers when examining the effect of amino acid substitution on protein structure and any potential interaction(s) with surrounding residues.

Since submission of our study, another crystal structure of hPLD3 was published by Roske *et al*. [29] and mouse PLD3 and human PLD4 crystal structures have been published by Yuan *et al*. [30]. All three PLD3 structures are very similar, albeit there are differences in flexible loops (Fig. 2). We also find that hPLD3 and hPLD4 structures are very similar, with slight differences observed in the length and orientation of some α‐helices and β‐strands and the conformation of flexible loops (Figs 6, 7, 9). Both groups also determined structures in the presence of ssDNA which allowed a detailed mechanism for the nuclease activity to be postulated [29, 30]. Briefly, they find that PLD3 likely uses a ping‐pong mechanism where H416 attacks the phosphodiester bond to form a covalent intermediate, followed by product release aided by H201.

Our hPLD3 structure resembles the previously determined hPLD1/2 structures [33, 34] despite pairwise sequence identities to hPLD3 of < 14%. There is a conservation of secondary structure between the respective cores (Fig. 6A) and the spatial locations of the catalytic histidine and lysine residues overlap closely (Fig. 6B). However, notable differences between hPLD3 and hPLD1/2 were observed. For example, features seen in hPLD1/2, such as a helix ‘arc’, a hydrophobic ‘lid’ and a polybasic motif, are not observed in hPLD3 (Fig. 7) and the entrance to the catalytic site is much larger in hPLD3 (Fig. 6C).

Only three members of the human PLD family, including hPLD1 and hPLD2, have been shown to exhibit phospholipase activity. Whether hPLD3 is a phospholipase has been controversial [14, 19, 20, 21, 22, 23]. There is circumstantial evidence suggesting the enzyme might not be a phospholipase; for example, it exhibits very low sequence identity with hPLD1/2 and possesses a non‐canonical catalytic sequence motif. In our own analysis, we have found a number of distinguishing features between hPLD1/2 and hPLD3 as noted above. Here we showed that highly purified enzyme does not exhibit any measurable phospholipase activity under the conditions tested but instead has nuclease activity (Fig. 8). We cannot exclude that hPLD3 might require co‐factors such as metals for phospholipase activity although hPLD1/2 does not require co‐factors for this activity.

Our results indicate that our hPLD3 construct exists in a monomer‐dimer equilibrium in solution (with the possibility of minor higher‐order oligomers). Around neutral pH, there are equal proportions of monomers and dimers whereas dimers are favored at low pH (~ 80% at pH 4.5). The recently published hPLD3 structures appear dimeric but all have been crystallized and analyzed at low pH [29, 30]. The physiological relevance of the oligomeric state is not clear as the catalytic sites are far apart in the dimer structures but possibly contribute to stability of the protein in the low pH environment of the lysosome. hPLD3 is a type II transmembrane protein with a short cytoplasmic tail but there is no evidence to date that it transduces signals across the membrane (which might be facilitated by a monomer‐dimer equilibrium by analogy with cytokine receptors).

We have mapped published AD risk variants onto the crystal structure of hPLD3 to seek molecular insights into whether they are likely to have functional effects (Fig. 5). V232M is the only SNP directly associated with AD [1, 2, 3, 4, 38]. The V232M substitution has been shown to impair O‐glycosylation at pT271, a post‐translational modification essential for normal trafficking and localization, leading to enlarged lysosomes. However, in the PLD3 crystal structure, these residues are far apart with a distance of over 21 Å. It seems likely from our analysis that most of the substitutions could destabilize the PLD3 structure.

Overall, the results presented here provide an increased understanding of PLD3 biology and will aid the development of AD therapeutic agents targeting the enzyme.

## Materials and methods

### Protein expression and purification

Bioinformatic tools were used to predict the secondary and tertiary structures of hPLD3 and then utilized to design a variety of hPLD3 constructs focused on the luminal domain. The bioinformatics analysis of the canonical hPLD3 sequence (UniProt ID: Q8IV08) was conducted using: (a) Quick2D [43] and Phyre2 [44] to predict the secondary structure elements, and (b) a deep learning distance‐based protein folding version of RaptorX [45] and AlphaFold2 [46] to predict the tertiary structure. For mammalian expression of hPLD3, the DNA fragment encoding an N‐terminal secretion signal peptide, followed by a hexa‐His tag, then an eight residue linker, a TEV cleavage site and the luminal domain of hPLD3 from residues N71 to L490 (MESQTQVLMSLLFWVSGTCG‐HHHHHH‐SSGVDLGT‐ENLYFQS‐hPLD3 N71‐L490) was synthesized and subcloned into the pcDNA3.1(+) expression vector by GenScript (Piscataway, NJ, USA). The N‐terminal signal peptide (MESQTQVLMSLLFWVSGTCG) ensures the protein is transported out of the cells and the hPLD3 protein is then purified from the medium. The DNA sequence of hPLD3 isoform 1 was obtained from the UniProtKB/Swiss‐Prot protein sequence database (UniProtKB: Q8IV08, PLD3_HUMAN) (UniProt, 2021). The PureLink HiPure Expi Plasmid Megaprep Kit (Invitrogen, Waltham, MA, USA) was used to prepare a large quantity of transfection‐grade plasmid DNA by following its User Guide (Publication number K210008XP).

Firstly, 200 mL of Expi293 GnTI‐ cells (Invitrogen, Waltham, MA, USA) in Expi293™ expression medium at 3.0 × 10^6^ were transfected following the User Guide (Publication number MAN0007814). The final DNA concentration of the transfection was 1 μg⋅mL^−1^. 20 h post‐transfection, ExpiFectamine 293 Transfection Enhancers 1 and 2 were added as per the User Guide and then Lupin Peptone (Solabia, PANTIN Cedex, France) was added to a final concentration of 0.5%. The cells were grown post‐transfection in vented flasks at 110 rpm, 37 °C, 8% CO_2_ for 5 days in total.

Culture supernatants were collected and hPLD3 was purified by affinity chromatography on a 5 mL HisTrap excel column (Cytiva, Marlborough, MA, USA) in buffers containing 20 mm Tris pH 7.5, 250 mm NaCl and 500 mm imidazole. This was followed by size exclusion chromatography (16/60 Superdex 200, Cytiva, Marlborough, MA, USA) in 20 mm Tris pH 7.5, 250 mm NaCl. The protein expressed at very high yield of 55 mg⋅L^−1^ and it was concentrated to 5.9 mg⋅mL^−1^ and frozen at −80 °C.

**Scheme 1:**
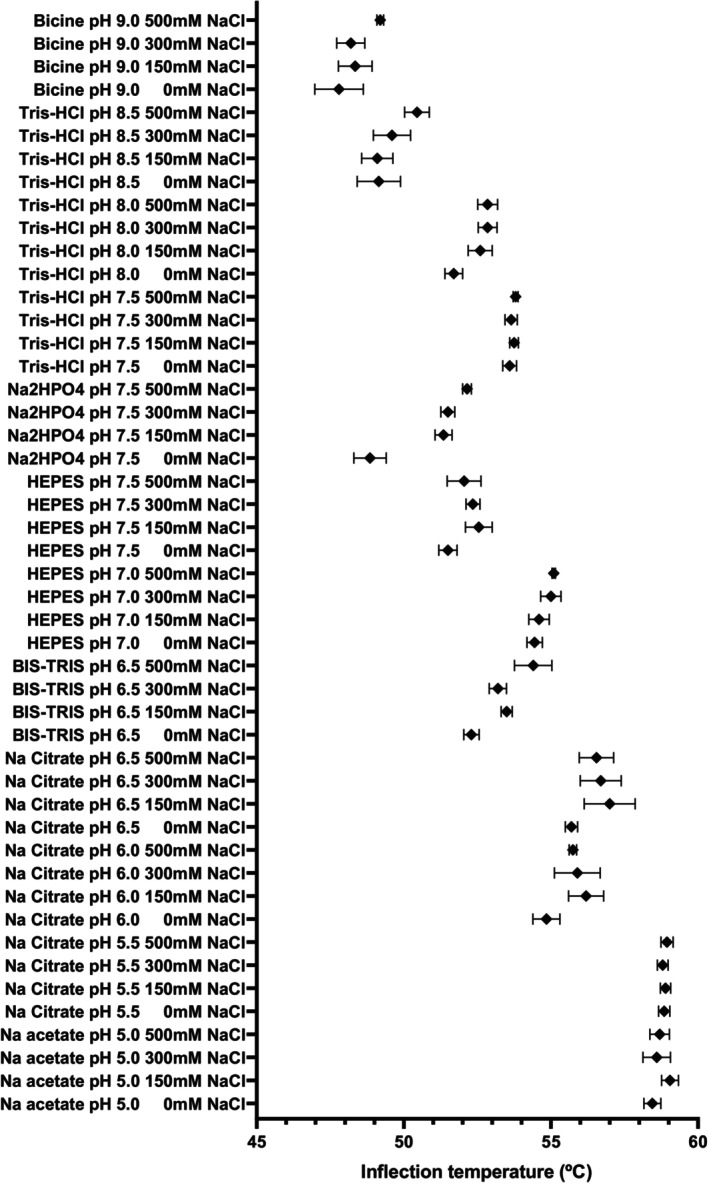
DSF analysis to determine the optimum storage buffer conditions for hPLD3. The mean ± SEM of the inflection temperature (°C) for each buffer condition (*n* = 4) is shown. The inflection temperature of hPLD3 appears to be dependent on the type and pH of the buffer. hPLD3 was thermodynamically stable under acidic buffer conditions (pH 5.0–5.5).

The optimal buffer for storage was determined by DSF (Scheme 1) and intrinsic fluorescence (Tycho; NanoTemper, Munich, Germany). The optimal storage buffer for hPLD3 was an acidic buffer of sodium acetate or sodium citrate at pH 5–5.5.

### Dynamic light scattering (DLS)

Purified hPLD3 in 20 mm Tris pH 7.5 and 250 mm NaCl was diluted to 0.63 mg⋅mL^−1^ in a 1/10 dilution in each of the buffers listed in Table 3. Samples were spun down for 1 min at 1000 rpm and incubated on ice for 1 h. Prometheus Panta (NanoTemper) was used to run all DLS measurements. High sensitivity capillaries (NanoTemper) were filled with ~10 μL of each sample and the dynamic light scattering (DLS) measurements were performed at 25 °C. The assay setup was performed on the Prometheus Panta Control software (NanoTemper), while analysis of the results was conducted on the Prometheus Panta Analysis software (NanoTemper). Each sample was measured in triplicate and within each group 10 DLS measurements run per replicate. The size distribution graphs represent a sum of all the DLS scans performed per condition, by averaging iterations of all replicates, layering them on top of each other, and finally reporting the hydrodynamic volume of the averaged peak. To calculate the hydrodynamic volume of the hPLD3, our structure file in PDB format was amended accordingly to provide information relating to monomeric or dimeric protein. HullRad algorithm [47] calculated the monomeric hPLD3 at ~3.1 nm and the dimeric hPLD3 at ~4.3 nm.

**Table 3:** Composition of buffers used for the characterization of hPLD3 (i.e. DLS, native page, mass photometry).

Buffer A	20 mm Tris pH 8.5, 250 mm NaCl
Buffer B	20 mm Tris pH 7.5, 250 mm NaCl
Buffer C	20 mm sodium citrate pH 6.5, 250 mm NaCl
Buffer D	20 mm sodium citrate pH 5.5, 250 mm NaCl
Buffer E	20 mm sodium acetate pH 4.5, 250 mm NaCl

### Native gel analysis

Purified hPLD3 in Buffer B (Table 3) at 2.5 μg was mixed with the Novex™ Native Tris‐Glycine Sample Buffer (Invitrogen) and resolved by a NuPAGE 3–8% Tris Acetate gel (Invitrogen) in a Novex Native Tris‐Glycine running buffer (Invitrogen) for 2 h. NativeMark unstained protein standard (Invitrogen) was applied as a known molecular weight ladder. An in‐house produced soluble protein with known molecular weight (30 kDa) was also prepared as the hPLD3 sample and used as an additional molecular weight control. The gel is representative of two biological repeats.

### Mass photometry

All mass photometry measurements were performed on the TwoMP instrument (Refeyn, Oxford, UK) at room temperature. In a typical experimental procedure, sample well cassettes (Refeyn) were fixed onto ready‐to‐use clean sample carrier slides (Refeyn). Initially a calibration was performed. A 10 μL droplet of buffer was applied into one well to establish correct focus prior to sample application. 10 μL of 25 nm BSA (Sigma‐Aldrich, A3311) was mixed thoroughly with the buffer droplet, reaching a final volume of 20 μL and final BSA concentration at 12.5 nm. 60 s movies were collected using the AcquireMP software (Refeyn, Oxford, UK). hPLD3 samples were measured following a similar procedure. 6.3 mg⋅mL^−1^ (128.3 μm) protein in Buffer B (Table 3) was diluted to 25 nm in two buffers filtered with 0.22 μm filter: Buffer B and Buffer E (Table 3). Serial dilutions were prepared for both buffer conditions and samples were incubated with at room temperature for ~20–30 min. The final hPLD3 concentrations measured were: 12.5, 6.25, and 3.125 nm. Raw data were processed using the DiscoverMP software (Refeyn, Oxford, UK). The calibration was applied to all measurements while the results were plotted as mass distribution histograms. All data shown represent single experiments (but are representative of two biological repeats).

### Protein crystallization and X‐ray data collection

Crystals of hPLD3 could only be grown with the hexa‐His tag left on. Initial crystallization trials were set up using the sitting‐drop vapor diffusion method, with sparse‐matrix screens, where each well contained 40 μL of well solution and 0.1 μL of well solution mixed with 0.1 μL of purified protein sample, which was dispensed using an in‐house NT8 protein crystallization robot (Formulatrix, Bedford, MA, USA). An automated imaging system – Rock Imager (Formulatrix) captured crystal drop images under visible light and UV fluorescence. Optimization was performed using the hanging drop vapor diffusion method with 24‐well Linbro style plates (Molecular Dimensions Ltd, Rotherham, UK), where each well contained 500 μL of well solution and 1 μL of purified protein sample mixed with 1 μL well solution on a 22 mm siliconized glass circular cover slide (Hampton Research, Aliso Viejo, CA, USA). The best crystals were grown at 20 °C in 0.17 m ammonium sulfate, 15% (v/v) glycerol and 25% (w/v) PEG 4000. Crystals were flash‐cooled in liquid nitrogen directly from crystallization drops. X‐ray diffraction data were collected at 100 K using the MX2 beamline at the Australian Synchrotron, part of ANSTO, and made use of the Australian Cancer Research Foundation (ACRF) detector and Blue‐Ice software [48]. X‐ray data collection statistics are listed in Table 1.

### X‐ray data processing and structure refinement

Diffraction data were indexed, integrated and scaled using *XDS* [49], analyzed using *POINTLESS* and merged using *AIMLESS* from the *CCP4* suite [50]. Initial phase estimates were obtained using molecular replacement with *MOLREP* in *CCP4* with an AlphaFold2 [46] model (AF‐Q8IV08‐F1‐model_v4.pdb) as the search probe. Two molecules were located in the a ASU. Refinement was performed using *REFMAC* from *CCP4* and *phenix.refine* [51], with iterative model‐building performed in *Coot* [52]. The final *R*_work_ and *R*_free_ were 0.185 and 0.209, respectively (Table 1). The Ramachandran plot indicated that all residues of the model lay within energetically favorable regions. Within the asymmetric unit there are two PLD3 molecules, 9 sulfate ions, 3 glycerol molecules, and a total of 387 water molecules; the two PLD3 molecules in the asymmetric unit are each comprised of 416 amino acid residues (Q72‐L490) and two oligosaccharides. Notably, three residues, A98 to T100, were not modeled due to weak electron density. Refinement statistics are tabulated in Table 1.

### *In silico* modeling of AD risk variants, stability predictions and visualization

Models for individual AD risk variants (I163M, V232M, N284S, C300Y, R356H and P410S) were constructed by *in silico* mutation of the native residue in the hPLD3 crystal structure, followed by geometry optimization, using the modeling program SYBYL‐X 2.1.1 (Certara, L.P., https://www.certara.com). An alternative low‐energy side‐chain conformation from a conformer library within the Biopolymer module of SYBYL‐X 2.1.1 was used to replace any clashing amino acid side‐chains. Each mutant PLD3 model was geometry optimized using the conjugate gradient minimization method, molecular mechanics MMFF94s force field and partial atomic charges for a maximum of 5000 iterations or until the gradient of successive iterations was <0.05 kcal⋅mol^−1^⋅Å (all other parameters were at default values).

MUSTANG version 3.2.3 [53] was used to align human PLD1, PLD2, PLD3 and PLD4 crystal structures via their secondary structure elements. Solvent accessible volumes and depths of the catalytic site funnel‐shaped cavity for the four human PLDs were calculated using the web‐based implementation of the parKVFinder software KVFinder‐web (https://kvfinder‐web.cnpem.br/) [54]. A probe diameter of 1.4 Å, a removal distance of 2.6 Å and a volume cutoff of 500 Å^3^ were used for the KVFinder‐web calculations. The stability of the AD risk variants (change in Gibbs free energy, ΔΔG^stability wt>mt^) was predicted using DDMut (https://biosig.lab.uq.edu.au/ddmut/) [39]. The APBS Electrostatics Plugin tool for PyMOL was used to calculate the electrostatic potential surface in the range −3 kT/e to +3 kT/e. The PyMOL Molecular Graphics System version 2.1.1 Schrodinger, LLC (https://pymol.org) was used for visualization and preparing the structural images.

### 5′ exonuclease activity assay

Exonuclease activity was measured using the End‐labeled Fluorescence‐Quenched Oligonucleotide (EFQO) assay developed by Cappel *et al*. [28]. Serial dilution series of recombinant hPLD3 and PLD from *Streptomyces chromofuscus* (Sigma Aldrich, North Ryde, Australia) in 50 mm MES pH 5.5, 50 mm NaCl buffer containing 1 pmol⋅μL^−1^ FAM‐ssDNA‐substrate (Biomers, Ulm, Germany) (Sequence: ACCATGACGTTCCTGATGCTAAGTATG*C* A*C* with * indicating a phosphorothioate bond) were loaded into 96 well trays to a final volume of 100 μL in triplicate. After 2 h incubation at 37 °C, activity was measured using an EnSpire Multimode Plate Reader (PerkinElmer, Glen Waverley, Australia) at excitation: 485 nm/emission: 530 nm. A substrate control without protein and a protein control without substrate were measured together with the samples. Background fluorescence correction was performed by subtracting the mean fluorescence intensity of the no‐protein substrate controls from each data point.

### Phospholipase activity assay

Phospholipase activity was measured using the Amplex Red PLD Assay Kit (ThermoFisher Scientific, Scoresby, Australia) following the manufacturer's instructions. Serial dilution series of recombinant hPLD3 and PLD from *Streptomyces chromofuscus* (Sigma Aldrich, North Ryde, Australia) were loaded into 96 well trays in duplicate. Assays were performed following the continuous assay method using the provided pH 8.0 reaction buffer with a 60 min incubation step at 37 °C with 0.25 mm phosphatidylcholine (lecithin) substrate solution. Assays were also performed using the two‐step PLD assay method, replacing the reaction buffer for recombinant hPLD3 with 25 mm MES pH 5.0, 2.5 mm CaCl_2_, and incubating the first‐step reactions for 2.5 h at 37 °C with 0.5 mm phosphatidylcholine (lecithin) substrate solution. Results of the colorimetric assays were measured using an EnSpire Multimode Plate Reader (PerkinElmer, Glen Waverly, Australia) at excitation: 540 nm/emission: 590 nm. Background fluorescence correction was performed by subtracting the mean fluorescence intensity of no‐protein controls from each data point.

## Conflict of interest

The authors declare no conflict of interest.

## Author contributions

MWP, TLN, SJH, NCH, GANC and JHG designed the experiments. KI, SJH, MEG, TLN, NCH and GANC performed experiments and analyzed data. NCH and GANC supervised the biochemical studies and SJH and MAG supervised the crystallographic studies. SJH, MEG, TLN and MWP wrote the paper.

## Data Availability

Structure coordinates and structure factors have been deposited in the Protein Data Bank with the following accession code: 8V5T.
